# New-Onset Refractory Status Epilepticus Due to a Novel MT-TF Variant: Time for Acute Genetic Testing Before Treatment?

**DOI:** 10.1212/NXG.0000000000200063

**Published:** 2023-03-15

**Authors:** Elisabetta Indelicato, Johannes Pfeilstetter, Michael Zech, Iris Unterberger, Julia Wanschitz, Steffen Berweck, Sylvia Boesch

**Affiliations:** From the Center for Rare Movement Disorders Innsbruck (E.I., S. Boesch); Department of Neurology (E.I., I.U., J.W., S. Boesch), Medical University of Innsbruck, Austria; Hospital for Neuropediatrics and Neurological Rehabilitation (J.P., S. Berweck), Centre of Epilepsy for Children and Adolescents, Schoen Klinik Vogtareuth, Germany; Institute of Neurogenomics (M.Z.), Helmholtz Zentrum München; Institute of Human Genetics (M.Z.), School of Medicine, Technical University of Munich; and Department of Pediatric Neurology and Developmental Medicine Dr. von Hauner Children´s Hospital (S. Berweck), Ludwig Maximilian University of Munich, Germany.

## Abstract

**Objective:**

The gene MT-TF encodes the mitochondrial tRNA of phenylalanine (tRNA^phe^). Its variations have been described as extremely rare etiologies of a variety of mitochondrial phenotypes.

**Methods:**

By means of whole-exome sequencing (WES), we detected a novel likely causative MT-TF variant (m.610T>C) in a family presenting with a combined movement disorder and epilepsy phenotype. The variant was present at 97% heteroplasmy in the peripheral blood and in a homoplasmic state in skin fibroblast-derived DNA.

**Results:**

The inaugural manifestation in the index patient was new-onset refractory myoclonic status epilepticus (NORSE) at the age of 29 years. Her son presented later with developmental regression and myoclonic epilepsy. On the beginning of valproate because of ongoing myoclonic seizures, the index patient developed a generalized brain edema requiring bilateral craniotomy. In the course of the disease, epileptic manifestations abated, and both patients developed a severe movement disorder phenotype with prominent spastic-dystonic features. Both patients did not display any further sign of mitochondrial disease.

**Discussion:**

Our report expands the clinicogenetic background of tRNA^phe^ disease spectrum and highlights pitfalls in the diagnostics and management of mitochondrial epilepsy. The present findings advocate the introduction of rapid genetic testing in the diagnostic flow chart of NORSE in adults.

The gene *MT-TF* encodes the mitochondrial tRNA of the amino acid phenylalanine, and its variants have been occasionally described as rare etiology of typical mitochondrial syndromes.^1-4^

Herein, we report a family with a mitochondrial phenotype due to a novel point mutation (m.610T>C) in *MT-TF*, harbored in a homoplastic state. The clinical phenotype consisted of new-onset refractory myoclonic status epilepticus in the mother and developmental regression along with myoclonic epilepsy in her son. In the course of the disease, epileptic manifestations abated, and both patients developed a severe movement disorder phenotype with spastic-dystonic features. With this report, we expand the phenotype of *MT-TF*–related disorders and highlight pitfalls in the clinical diagnosis and management of mitochondrial disorders in adults.

Written informed consent was given by the legal guardians of both patients for the genetic testing and for the publication within a research project on rare movement disorders (Study nr. 1133/2018).

## Clinical Description

**Figure 1:**
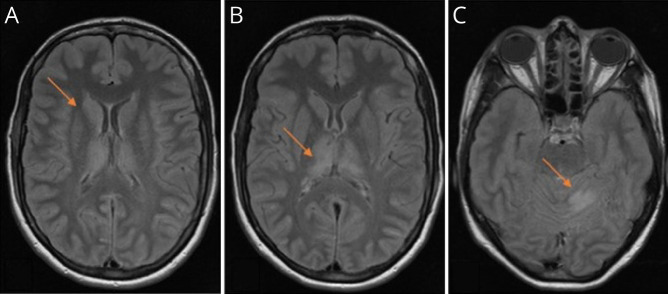
Brain MRI From the Index Patient MRI performed 12 days after symptoms onset, during an ongoing myoclonic status. Fluid-attenuated inversion recovery T2 sequences showing hyperintensities in the right N. caudatus (A) as well as in both thalami (B) and in the cerebellum (C), compatible with an ongoing status epilepticus.

**Figure 2:**
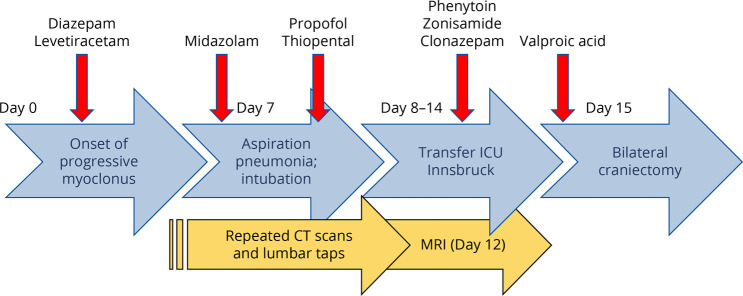
Graphical Representation of the Clinical Evolution in the Index Patient The key steps of clinical management and ASM trials within the first 15 days from the onset of disease are summarized. ASM = antiseizure medication.

The index patient had an unremarkable clinical history until the age of 29 years, when she suddenly developed myoclonic jerks of the trunk and the head. She received diazepam in an outpatient setting and was subsequently admitted to the local hospital. She developed aspiration pneumonia and rapidly deteriorated. She was intubated and transferred in the intensive care unit (ICU). Here, the clinical picture and the EEG were compatible with a status myoclonicus, which was refractory to the escalating therapy with levetiracetam, midazolam, and propofol. A burst suppression pattern was reached under thiopental, but attempts to reduce it were inevitably followed by status reoccurrence. Therefore, she was transferred to our university hospital. Brain MRI (see Figure 1) and repeated CSF analysis remained inconclusive. MRI spectroscopy showed an overall reduction of N-acetylaspartate peak but no lactate peak. Because of refractory status despite the multiple antiseizure medication (ASM) trials (including levetiracetam, phenytoine, zonisamide, and clonazepam), valproate was started. Under valproate, she developed encephalopathy (ammonium level 66,5 µmol/L; range 11–48 µmol/L) with a generalized brain edema, which did not improve on conservative therapy and eventually led to bilateral craniectomy (for a graphical summary until this time point see Figure 2). After craniectomy status epilepticus shortly abated, but the patient went on experiencing several seizures daily, including recurrent myoclonic status epilepticus. Short seizure-free phases were invariably followed by recrudescences. She additionally developed recurrent metabolic derangements on other ASMs (severe metabolic acidosis on topiramate and transaminase elevation along with EEG signs of metabolic encephalopathy on zonisamide, rufinamide, and barbexaclone). After the first ICU stay (6 months), the patient displayed clinically an encephalopathic state with fluctuating vigilance and a spastic tetraparesis. In the following years, she showed a clinical improvement with the regain of communication skills while motor functions remained severely impaired.

The index patient had a 3-year-old healthy child at the time of onset of disease. The boy (Pt. 2) had an unremarkable clinical history until the age of seven years when, following pneumonia, he showed a sudden worsening of motor functions. Free walking or sitting were no longer possible, and he could no longer speak. Repeated MRI and blood and CSF diagnostics were unremarkable. Two months later, he developed myoclonic status epilepticus, which promptly abated on intravenous midazolam. Under oral ASM (currently levetiracetam/oxcarbazepine), he remained seizure free up to now. However, his motor skills did not recover completely, and currently, at the age of 17 years, he displays a generalized spastic-dystonic movement disorder with severe dysarthrophonia. Cognition, understanding, and communication skills are normal.

### Additional Studies

Muscle histology in Pt.2 (M. Vastus lateralis) showed slight nonspecific myopathic changes, but no ragged-red fibers and a normal cytochrome-C oxidase staining. Respiratory chain enzymes analysis was unremarkable. Nerve conduction studies, EMG, ECG, echocardiography, ENT, and ophthalmologic evaluation including fundoscopy yielded normal findings in both patients. Renal function tests were within the norm in both patients in repeated referrals. An initial transitory plasma lactate elevation in the index patient was compatible with a septic state during pneumonia.

### Genetic Studies

Whole-exome sequencing (WES) using peripheral blood-derived DNA was performed on the affected mother-son pair and the son's healthy father as described in detail before.^5^ Analysis of nuclear-encoded genes associated with the affected individuals' phenotypes did not yield any pathogenic or likely pathogenic variants. Assessment of variants in mitochondrial DNA using previously reported in-house–established methods^6^ revealed a rare alteration in *MT-TF* (NC_012920.1): m.610T>C in the affected mother and her son. This variant has not been previously reported, and it was absent from 20,000 in-house control exomes. In peripheral blood, the variant was present at 97% heteroplasmy in both patients (with 29× and 33× coverage of the position in mitochondrial DNA, respectively). Validation in a second biospecimen was undertaken, showing that the *MT-TF* variant was present in a homoplasmic state (100% of 42 reads) in skin fibroblast-derived DNA from the son.

### Data Availability

Further clinical data are available on request.

## Discussion

Variants at the *MT-FT* locus are far rare genotypes, with only 16 pathogenic variants described up to date (see Table).^7^

**Table:**
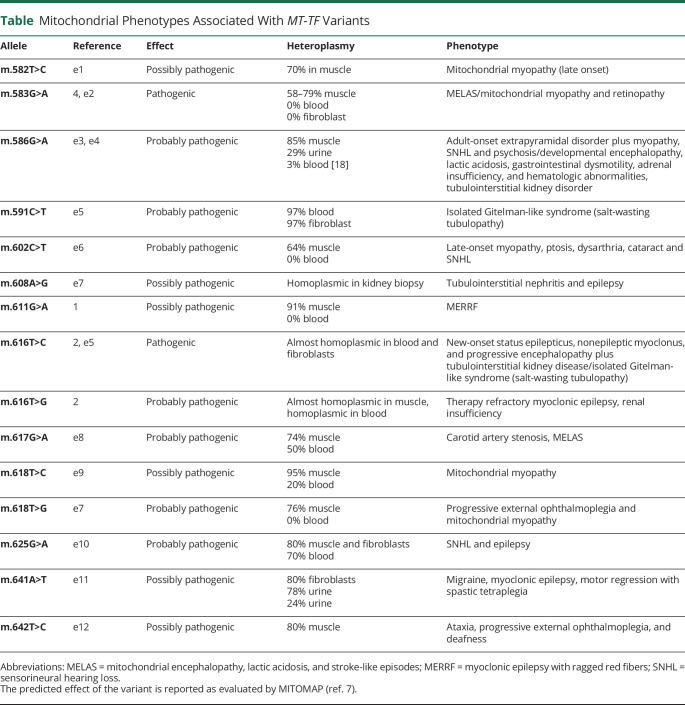
Mitochondrial Phenotypes Associated With *MT-TF* Variants

In the present family, a novel *MT-TF* variant became initially manifest with a new-onset progressive myoclonic epilepsy in a 29-year-old, otherwise healthy woman. The pathogenic mDNA variant was close to the homoplasmic state in the analyzed tissues in both affected family members. In both patients, the clinical phenotype expanded beyond classical features, such as therapy refractory myoclonic epilepsy, to reveal later in the disease course prominent movement disorders with spastic-dystonic components which currently represent the main symptom.

Although the initial phenotype in the index patients may suggest mitochondrial disease, she did not display other typical features (adult age and negative family history at disease onset, absence of any other biochemical or clinical hints^8^). For this reason, because of ongoing superrefractory status, valproate was applied as *extrema ratio*. Valproate is a first-line medication in myoclonic epilepsy, but in mitochondrial disorders should be used with caution.^9^ In our case, its administration likely triggered or precipitated a malignant brain edema that led to acute bilateral decompression craniotomy.

New-onset therapy refractory status epilepticus (NORSE) can result from several conditions, of which the most common are autoimmune encephalitis and infectious diseases.^10,11^ Mitochondrial diseases are listed as rare etiology of NORSE, and the current diagnostic recommendations do not contemplate a systematic screening for mitochondrial defects in adults,^10,12,13^ which is still time and resource consuming outside of specialized centers. Since a normal CSF examination rules out the most common differential diagnoses of NORSE, the application of a rapid genetic test, as further diagnostic step, would have been a posteriori the approach of choice in the index patient. Rapid genetic testing strategies have been successfully applied in the setting of fulminant pediatric disorders, opening a window of opportunity when a potentially treatable disorder is untangled.^14,15^ In our case, the time pressure because of unsuccessful status treatment and the concern of omitting a potentially effective drug, motivated, in the setting of missing guidelines, a difficult therapeutic choice. It remains speculative whether an earlier diagnosis, by means of rapid genetic testing, could have influenced the course of the disease in our patient. However, it would have prevented complications due to inappropriate application of first-line pharmacologic therapies (valproate in the setting of myoclonic epilepsy).

This report highlights the utility of WES in unravelling rare mitochondrial disorders. It moreover points out the need to implement guidelines for acutely screening a potential mitochondrial defect in adults in case of a marginal clinical suspicion and immanent therapeutic implications.
